# Age-Related Changes in Expectation-Based Modulation of Motion Detectability

**DOI:** 10.1371/journal.pone.0069766

**Published:** 2013-08-09

**Authors:** Theodore P. Zanto, Robert Sekuler, Chad Dube, Adam Gazzaley

**Affiliations:** 1 Department of Neurology, University of California San Francisco, San Francisco, California, United States of America; 2 Departments of Physiology and Psychiatry, University of California San Francisco, San Francisco, California, United States of America; 3 Volen Center for Complex Systems & Department of Psychology, Brandeis University, Waltham, Massachusetts, United States of America; University of Texas at Dallas, United States of America

## Abstract

Expecting motion in some particular direction biases sensitivity to that direction, which speeds detection of motion. However, the neural processes underlying this effect remain underexplored, especially in the context of normal aging. To address this, we examined younger and older adults' performance in a motion detection task. In separate conditions, the probability was either 50% or 100% that a field of dots would move coherently in the direction a participant expected (either vertically or horizontally). Expectation and aging effects were assessed via response times (RT) to detect motion and electroencephalography (EEG). In both age groups, RTs were fastest when motion was similar to the expected direction of motion. RT tuning curves exhibited a characteristic U-shape such that detection time increased with an increasing deviation from the participant's expected direction. Strikingly, EEG results showed an analogous, hyperbolic curve for N1 amplitude, reflecting neural biasing. Though the form of behavioral and EEG curves did not vary with age, older adults displayed a clear decline in the speed of detection and a corresponding reduction in EEG N1 amplitude when horizontal (but not vertical) motion was expected. Our results suggest that expectation-based detection ability varies with age and, for older adults, also with axis of motion.

## Introduction

**Figure 1:**
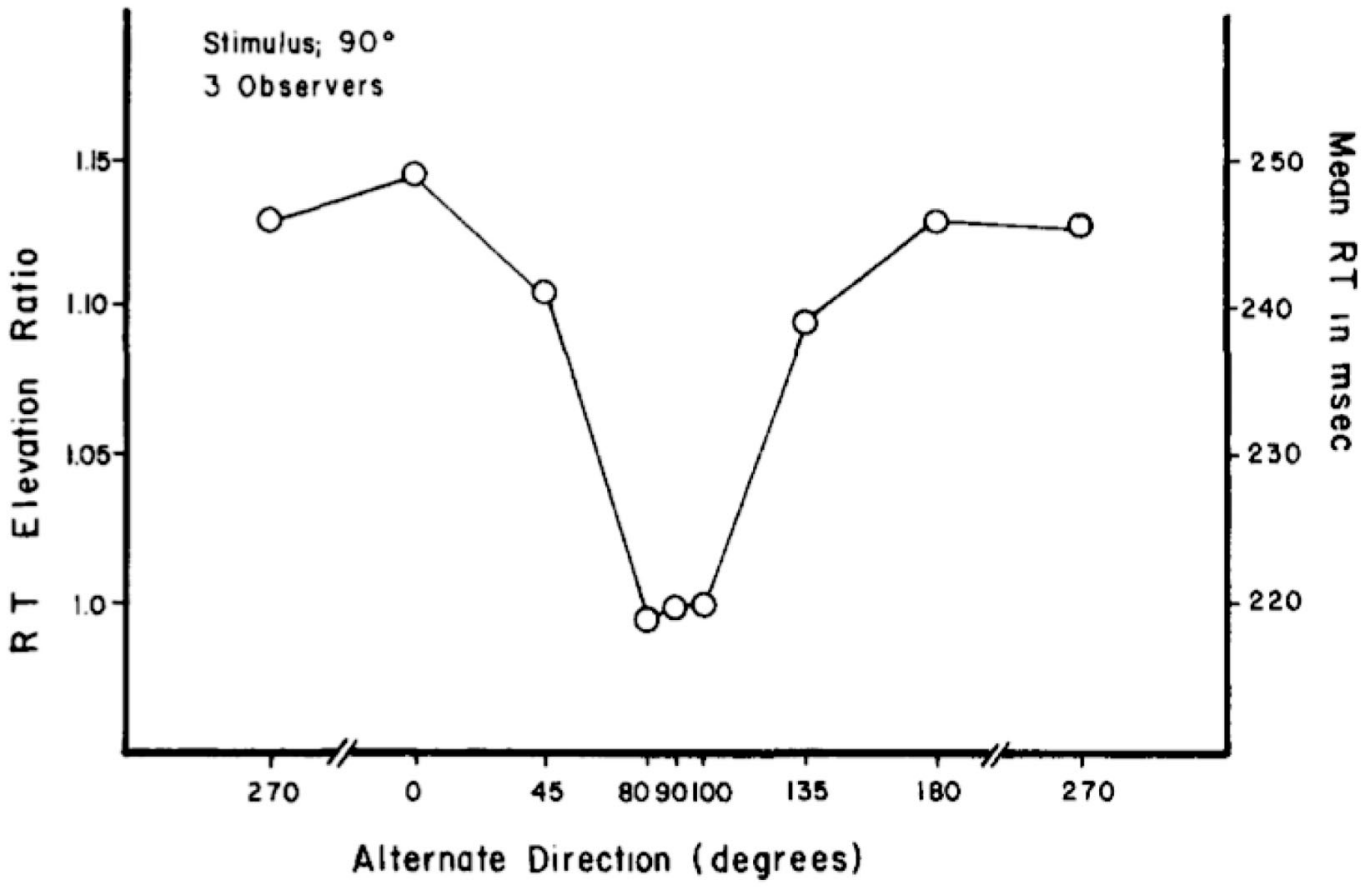
RT tuning curves from Ball and Sekuler (1980, Exp.1). The figure plots detection time for onset of an upward-moving (90°) stimulus in several conditions of uncertainty about the likely direction of motion on each trial. Uncertainty in this case was defined by the degree of angular separation between the 90° stimulus and the other direction of motion that might appear on a given trial. The y-axes plot RT and RT elevation ratio. The latter is the ratio of detection time to the 90° stimulus in each uncertainty condition, to the detection time for that same stimulus in a ‘certain’ condition where no other direction was presented on any trial.

It is well established that detection may be enhanced by expectations about an impending stimulus [1], [2], [3]. In the visual domain, Ball and Sekuler [4] (see also [3]) showed that reaction time (RT) to detect motion onset increased with subjects' uncertainty about the likely direction of motion on a given trial (Figure 1; reprinted from [4]). Their psychophysical approach was motivated by a neural biasing hypothesis, which was supported by their (purely behavioral) data: subjects' expectations involve selective activation of neural populations that are maximally sensitive to a particular range of motion directions.

Ball and Sekuler's study suggests that expectation for a specific direction of motion will yield speeded responses as 1) the probability of observing an expected direction increases (probability based expectation) and 2) the direction of motion becomes closer to an expected direction (directional tuning based expectation). Furthermore, their neural-biasing hypothesis suggests that expectation biases brain mechanisms that process motion stimuli. More specifically, the hypothesis implies that expectation for direction of motion enhances detection performance [5] by means of directional tuning of motion sensitive cortex [4]. Although broad support exists for a version of this neural biasing hypothesis [4], Ball and Sekuler did not complement their psychophysical data with corresponding neural data, leaving their interpretation of RT tuning curves open to question. Additionally, it is not known whether neural biasing is constant throughout the lifespan. The possibility that such biasing might be age-dependent is important because it could impact older adults' ability to extract and use the motion based neural information on which locomotion and navigation depend [6].

We have previously shown that healthy older adults do not always utilize predictive information to enhance performance during visual attention and working memory tasks [7], [8], [9], which is consistent with the hypothesis that cognitive aging entails some deficit in the use of probability-based expectations. Furthermore, older adults exhibit declines in motion detection and discrimination performance [10], [11], and single cell recordings in non-human animals as well as human neuroimaging studies show broadened tuning curves and less precise attribute- and category-specific coding in the posterior cortices of older brains [reviewed in: 12]. Together, these findings indicate that normal age-related changes include deficits in both probability based expectation and motion selectivity. Hence, we hypothesized that older adults would not exploit probability based or directional tuning based expectation processes to bias motion-selective neural regions and enhance detection performance as effectively as would younger adults. Specifically, we hypothesized that younger, and not older, adults would exhibit speeded motion detection response times when 1) the probability of observing the expected direction is increased (probability based expectation) and 2) when the direction of motion is expected or close to the expected direction (directional tuning based expectation).

Beyond these basic hypotheses, it is important to consider potential effects of directional specificity that may vary with age. Specifically, previous work suggests that perception of horizontal motion requires interhemispheric integration whereas vertical motion perception relies on intrahemispheric integration [13], [14], [15]. The additional distance neural signals must travel during interhemispheric processing implies greater age-related declines in horizontal than vertical motion processing, should the structural integrity of white matter tracts break down with age. As it is well established that these tracts do degrade with age [16], [17], we hypothesized that older adults would exhibit a decline in motion detection performance that is exacerbated when expecting motion along the horizontal axis.

To test these three hypotheses, we compared younger and older adults' performance in a motion detection experiment. Performance was assessed via mean response time (RT), and with an exponential Gaussian (Ex-Gaussian) analysis of RT distributions. The Ex-Gaussian analysis was meant to sharpen our understanding of which aspects of individuals' RT distributions were responsible for changes in mean RT [18], and has been previously used to assess age-related changes that may not be characterized by a standard assessment of mean RT [19], [20]. To test Hypothesis 1, we manipulated the probability of observing an expected direction of motion. This produced two conditions: Unidirectional, where stimuli across all trials moved in the same (expected) direction, and Multidirectional, where stimuli in half the trials moved in the same (expected) direction as the Unidirectional condition and the other half moved in a quasi-random (deviating) direction. Thus, probability based expectations were assessed via comparisons between conditions where stimuli moved in the expected direction 100% of the time versus 50% of the time. It was hypothesized that both age groups would exhibit speeded RT when the probability of observing an expected direction was 100% (Unidirectional condition) compared to 50% (Multidirectional condition), but that this RT gain would be greater for younger adults. To test Hypothesis 2, we compared detection performance between expected and deviating directions of motion within the Multidirectional condition. We hypothesized that younger, and not older, adults' RTs would quicken as the deviating direction approached the expected direction. To test Hypothesis 3, we presented the expected direction in either the vertical or horizontal axis of motion. We hypothesized that older adults would show a selective decline in motion detection when expecting horizontal, compared to vertical, motion.

To assess the neural mechanisms underlying age-related changes in motion-based expectation, we recorded each participant's electroencephalograph during the motion detection task. Analyses of neural data focused on early measures of visual processing, the P1 and N1 components of the event-related potential (ERP), which occur approximately 100 ms and 170 ms after motion onset, respectively. These components were chosen because they are modulated by attention to motion stimuli and have been localized to include the motion selective cortical region V5/hMT [21], [22]. Moreover, the N1 may be enhanced by deviations from expected stimulus features [23], suggesting it could serve as a neural marker for motion expectation. Finally, our combination of N1 and psychophysical analyses of motion detection provides an evaluation of previous behavioral work, which posited a correspondence between motion detection RTs and the sensitivity profiles of directionally-tuned neural mechanisms [4]. This is important, as a correspondence between RT tuning curves and changes in the N1 would greatly reinforce the interpretations of both measures.

## Methods

### 1. Participants

Twenty healthy young adults (mean age, 25 years; range, 22–32 years; 10 female) and twenty healthy older adults (mean age, 69 years; range, 64–79 years; 11 female) gave written informed consent to participate in the study, which was approved by the Committee on Human Research at the University of California San Francisco. All participants had normal or corrected-to-normal vision and were free of any ocular disease. Participants were screened to ensure they were healthy, had no history of neurological, psychiatric, or vascular disease, were not depressed, were not taking any psychotropic or hypertensive medications, and did not have strabismus, amblyopia, or an uncorrected astigmatism. Visual acuity was checked for each participant using a Snellen chart and corrective lenses were allowed when necessary to achieve 20/40 or better acuity. Additionally, all participants were required to have at least 12 years of education.

To ensure comparability with their age-matched peers, participants in the older age group were required to score within two standard deviations of published control values on 18 neuropsychological tests (Table 1). The neuropsychological evaluation consisted of tests designed to assess general intellectual function (MMSE [24]), geriatric depression (GDS [25]), verbal learning (CVLT-II [26]), visual reproduction (I & II, WMS-R [27]), logical memory (IA & IB, WMS-R [27]), working memory (symbol span, WMS-IV [28]; digit symbol and letter-number sequencing, WAIS-IV [29]), visual-motor sequencing (Modified Trail Making Test A & B [30]), phonemic fluency (F-A-S Test [31]), semantic fluency (animals [32], [33]), executive control (Stroop Interference Test [30], [34]), reading ability (WRAT4 – Reading [35]), and motor speed (Grooved Pegboard Test - left & right hand [36], [37], [38]).

**Table 1:** Neuropsychological test performance.

	Neuropsychological Screening Tests	Mean (SEM)
**Global**	MMSE (max. 30)	29.4 (0.2)
	Geriatric Depression Scale (max. 30)	2.6 (0.4)
	CVLT Long Delay Free Recall (max. 16)	12.1 (0.7)
	Visual Reproduction I (max. 43)	35.0 (1.2)
	Visual Reproduction II (max. 43)	28.2 (1.8)
**Memory**	Logical Memory IA (max. 25)	17.3 (0.7)
	Logical Memory IB (max. 25)	16.0 (0.7)
	Symbol Span (max. 50)	25.2 (1.0)
	Letter-Number Sequencing (max. 21)	11.6 (0.4)
**Attention/**	Digit Symbol (120 sec.)	63.8 (3.6)
**Processing**	Modified Trailmaking Test A (max. 150 sec)	39.0 (2.8)
**Speed**	Modified Trailmaking Test B (max. 240 sec)	73.1 (4.8)
	Phonemic Fluency (words in 60 sec.)	48.6 (2.3)
**Executive**	Semantic fluency (words in 60 sec.)	23.6 (1.2)
**Function**	WRAT-4 (max. 55)	50.0 (1.0)
	Stroop Interference (time to complete in sec.)	59.3 (2.6)
**Motor Speed**	Grooved Pegboard – left (time in sec.)	85.8 (3.0)
	Grooved Pegboard – right hand (time in sec)	77.1 (1.5)

Values are presented as the mean and standard deviation in parentheses. All individual participant scores were within two standard deviations of published control values.

### 2. Stimuli and experimental procedure

**Figure 2:**
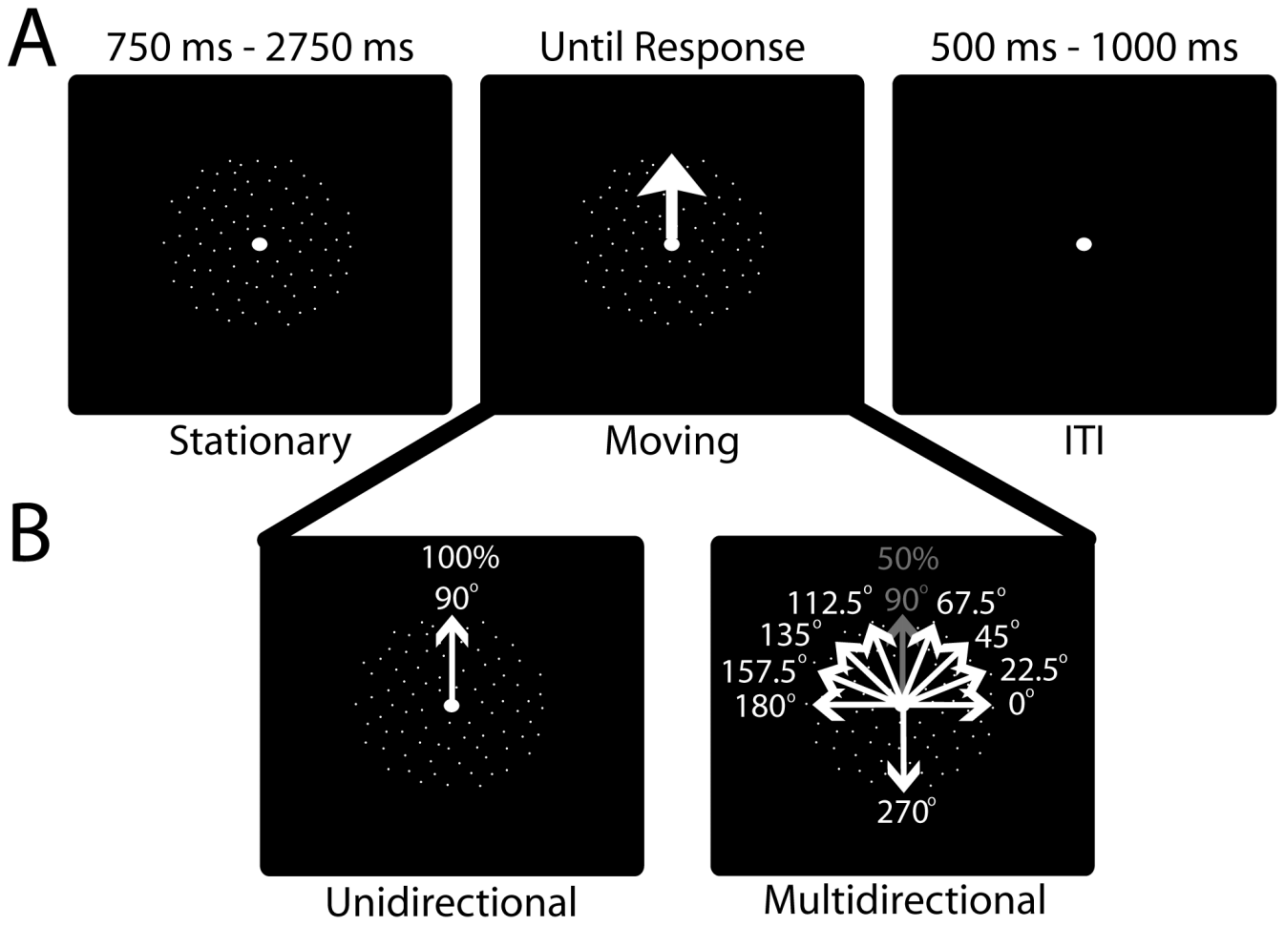
Experimental procedure. (A) Example of one trial. (B) Distribution of the directions of motion for each condition (if the main direction was up). Arrows indicate the direction of dot motion, and were not present during the experiment.

Stimuli were presented through Matlab (The Mathworks, Inc.), using the Psychophysics Toolbox extensions [39], [40], which were run on a Dell Optiplex GX620 computer with a 22″ Mitsubishi Diamond Pro 2040U CRT monitor. Participants sat in a dark room with heads supported by a chin rest 120 cm from the monitor. They were instructed to maintain fixation on a white disc (1° diameter) in the center of a black screen. Stimuli consisted of a circular aperture (8° diameter) within which spatially-random white dots were presented. During each trial (Fig. 2A), stationary dots were presented first, for a quasi-random interval (750 ms–2750 ms). Then, without warning to the participant, the dots began to move at 4° per second with 100% coherence. Participants were instructed to press a button with their right index finger as quickly as possible once the dots began to move. Following a button press, the moving dots disappeared and only the fixation disc remained for a quasi-random inter-trial interval (ITI; 500 ms–1000 ms).

Participants were randomly assigned one of four “expected” directions for stimulus presentation: up, down, left, or right. For analysis, the participants were grouped based on whether the expected direction of motion was vertical (up, down) or horizontal (left, right). The assigned expected direction was balanced across participants and age groups. The experiment consisted of two separate conditions: Unidirectional and Multidirectional. In the Unidirectional condition, the dots moved in the expected direction on every trial. In the Multidirectional condition, the dots moved in the expected direction on half of the trials, while deviations from the expected direction were presented for the other half of the trials (Fig. 2B). Although the 50% probability of observing the expected direction during the Multidirectional condition was consistent throughout the experiment, participants were not informed of this probability. The deviating directions consisted of 9 different directions, here expressed relative to the expected direction: ±22.5°, ±45°, ±67.5°, ±90°, and 180°. Thus, if a participant was assigned “up” as their expected direction in the Unidirectional condition, then using a coordinate system where 90° is up and 0° is to the right, the deviating directions presented during the Multidirectional condition would be: 0°, 22.5°, 45°, 67.5°, 112.5°, 135°, 157.5°, 180°, and 270°. Importantly, it should be noted that directions listed in degrees below are in reference to the expected direction, and not to a specific coordinate system. Each deviating direction had an equal probability of occurrence, presented randomly. Although participants were informed whether each condition was Unidirectional or Multidirectional at its outset, their task instructions remained the same: respond as quickly as possible to the onset of motion in any direction.

### 3. Data acquisition

Data were recorded during 18 blocks (6 Unidirectional and 12 Multidirectional) presented in a pseudo-randomized order (2 Multidirectional (M) blocks between 2 Unidirectional (U) blocks: U, M, M, U) and counterbalanced across participants. Participants had the option to pause halfway through each block to rest. Each block lasted approximately four minutes and consisted of 90 trials. The entire experiment lasted approximately 90 minutes and yielded 540 trials for the Unidirectional condition and 1080 trials for the Multidirectional condition. Of the 1080 trials in the Multidirectional condition, 540 were in the expected direction, 540 were in deviating directions (60 trials for each of the deviating directions).

Electrophysiological signals were recorded with a BioSemi ActiveTwo 64-channel EEG acquisition system in conjunction with BioSemi ActiView software (CortechSolutions, LLC). Signals were amplified and digitized at 1024 Hz with a 24-bit resolution and no online filter. All electrode offsets were maintained between ±20 mV.

### 4. Ex-Gaussian analysis

A change in mean RT across conditions, or a lack of change, depends heavily on the shape of the full RT distribution, and cannot in and of itself provide information about functionally important changes in an RT distribution's location or variance. Alternatives such as median or log-transformed RT also fail to provide information about distributional form. For instance, median RTs can show no change across conditions even though the distributions may differ greatly. An effect in log-transformed RTs, on the other hand, is difficult to interpret as the variable that is changing is not RT but log-transformed RT. Additionally, log-transforms are a way of allowing researchers to focus on changes in mean RT, potentially discarding or distorting effects on the spread of RT distributions that could be reliable and important. Ex-Gaussian modeling has been advocated as a solution to these problems, and will be used to supplement the standard analysis of mean RT as it provides a more fine-grained account of how the independent variable affect behavior [18]. The Ex-Gaussian distribution convolves a Gaussian and an exponential distribution, and is defined by three parameters: μ (mean of the Gaussian component), σ standard deviation of the Gaussian), and τ (mean of the exponential; larger values indicate a greater degree of skew in an RT distribution). The Ex-Gaussian model defines mean RT as the sum of the Gaussian and exponential means, and the standard deviation of RT is defined as √ (σ*^2^*+τ^2^). In order to isolate age-related differences in RTs' component processes, we fit an Ex-Gaussian function to each individual's RT distributions. In order to reduce the effects of contaminant RTs on distributional parameters, we removed RTs greater than ±2 SDs beyond a given subject's mean in a given condition prior to fitting, in line with criteria commonly used in Ex-Gaussian analyses [41]. We estimated Ex-Gaussian parameters for each individual, and in each condition of the analysis, using the SIMPLEX algorithm to minimize negative log likelihoods [42]. For this analysis, we deviated from the RT and ERP analyses in two ways. First, we excluded two younger subjects as they produced outlying values of σ and τ (though their μ components were similar to those of the remaining subjects, which supported their inclusion in the RT and ERP analyses). Second, we restricted the Uncertain Direction factor to two levels: the expected direction and the direction 180° opposite the expected direction. Otherwise, the relatively small number of observations in individual motion directions would have prevented the model's SIMPLEX search algorithm from converging for some subjects in the intermediate deviating directions.

### 5. EEG data analysis

All data were processed via custom Matlab scripts. Raw EEG data were referenced to the average off-line and band-pass filtered between 0.1 and 30 Hz with a zero-phase shift (non-causal) finite impulse response Butterworth filter. Data were segmented into epochs beginning 500 ms pre-motion onset and ending 700 ms post-motion onset. Epochs that exceeded a voltage threshold of ±75 µV were rejected. A 200 ms pre-cue baseline was subtracted from each epoch prior to calculating the ERP. Peak P1 values were chosen as the largest local peak amplitude between 50–150 ms post-stimulus onset, whereas the N1 was identified as the most negative local peak amplitude between 120–220 ms post-stimulus onset. These temporal windows do not reflect the range of observed peak latencies, but rather, serve to guide selection of ERP measures as previously reported [11], [43]. Mean amplitudes were measured by averaging over a 10 ms temporal window centered on each individual participant's peak prior to statistical analysis. Three posterior-occipital regions of interest were created by averaging over 4 electrodes from the central (OZ, IZ, O1, O2), left (P5, P7, P9, PO7), and right (P6, P8, P10, PO8) hemisphere, consistent with the topographical distribution of the visual ERP. Statistical analysis for EEG as well as behavioral data utilized a mixed design analysis of variance (ANOVA) with a Greenhouse-Geisser correction when appropriate. Post-hoc analyses consisted of two-tailed *t*-tests, corrected for multiple comparisons where appropriate via a false discovery rate procedure [44].

## Results

### 1. Response times

**Figure 3:**
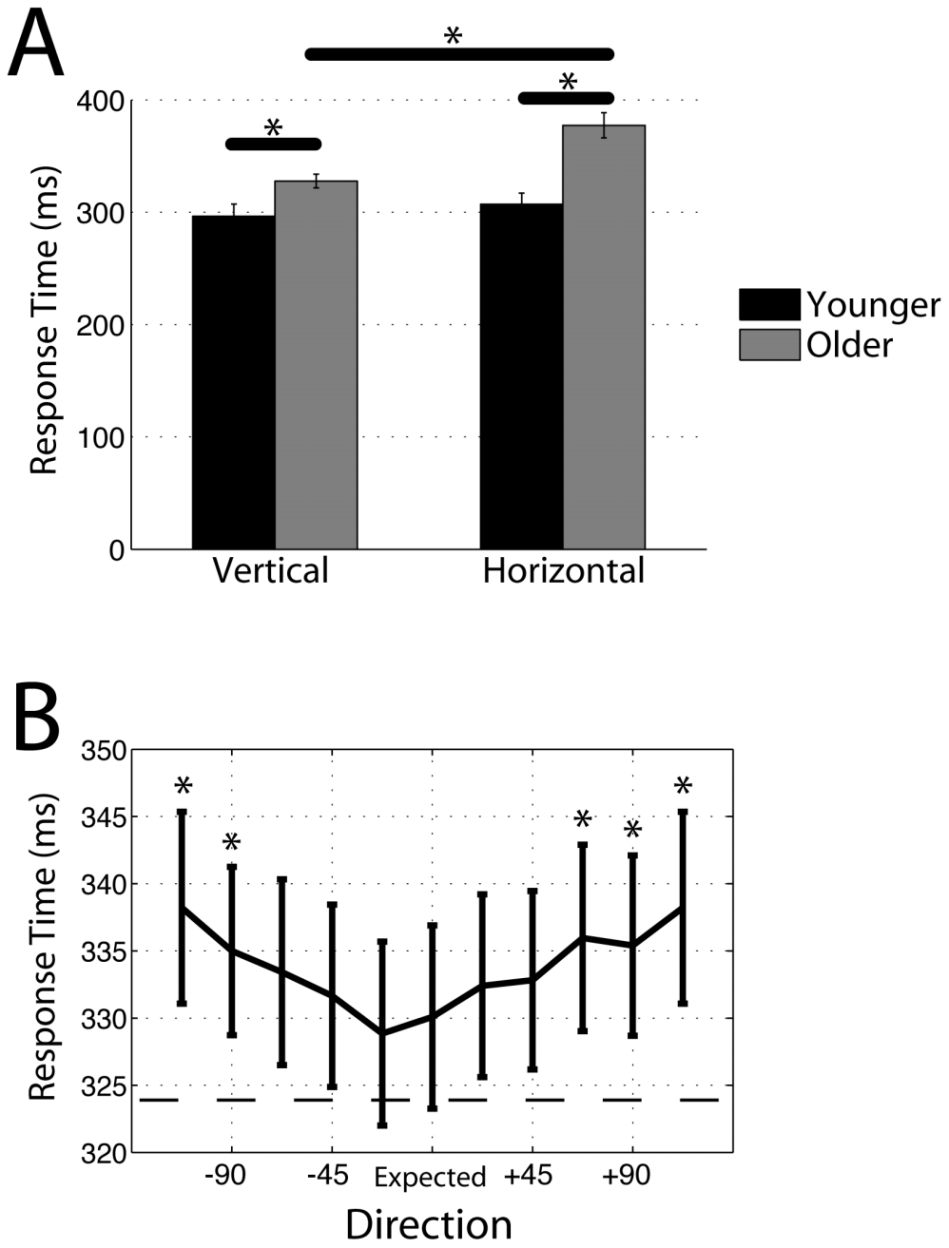
Response time results. (A) Age×Axis interaction (averaged across conditions) displays an age-related decline in horizontal motion detection. Asterisk indicates p<0.05. (B) Main effect of Deviation during the Mutlidirectional task. RT slows with increasing deviation from the Expected direction. Note: 180° was plotted on both sides for display purposes only. Dashed line depicts the average RT (averaged across Age and Axis) for the Unidirectional condition. Asterisk indicates p<0.05 (relative to Expected direction).

To assess probability based expectation influences on detection performance, response time (RT) to the expected directions of motion was compared across Unidirectional and Multidirectional conditions. Thus, RT data were submitted to an ANOVA with Age (Younger, Older), Probability (Unidirectional, Multidirectional), and Axis (Horizontal, Vertical) as factors. Main effects were observed for Age (F(1,36) = 27.34, p<0.01), Probability (F(1,36) = 19.76, p<0.01), and Axis (F(1,36) = 9.65, p<0.01), such that Younger adults (M = 302 ms, SEM = 5 ms) respond faster than Older adults (M = 353 ms, SEM = 6 ms), Unidirectional motion (M = 324 ms, SEM = 7 ms) elicits faster responses than Multidirectional motion (M = 330 ms, SEM = 7 ms), and Vertical motion (M = 312 ms, SEM = 5 ms) yields faster responses than Horizontal motion (M = 342 ms, SEM = 8 ms)(all p<.05). Additionally, we observed an interaction between Age and Axis (F(1,36) = 4.02, p = 0.05; Fig. 3A), indicating that Older adults are slower to respond to Horizontal motion than Vertical motion (t(18) = 3.88, p<0.01), but this was not the case for Younger adults (t(18) = 0.73, p = 0.47). Although direct comparisons between Age groups indicated Older adults were slower for each Axis of motion (p<0.05, each comparison), the Age×Axis interaction suggests that Older adults are disproportionally slowed in expectation for Horizontal motion detection. As Age did not interact with Probability, this suggests that both Younger and Older adults utilize probability based expectation processes to enhance motion detection abilities.

Although the previous analysis indicates a disproportionate age-related decline in horizontal motion processing, it remains unclear whether this is a decline in visual processing for the horizontal axis of motion, or expectation-related processes specific to that axis of motion. Therefore, directional tuning based expectation was assessed by submitting RT data from the different directions of motion in the Multidirectional condition to an ANOVA with Age (Younger, Older), Axis (expected Horizontal, expected Vertical), and Deviation (Expected, ±22.5°, ±45°, ±67.5°, ±90°, +180° (Note: these directions are not absolute, but relative to the expected direction; see methods for details)) as factors. Similar to the previous results, main effects were observed for Age (F(1,36) = 28.13, p<0.01) and Axis (F(1,36) = 7.48, p<0.01), such that Older adults (M = 359 ms, SEM = 6 ms) respond slower than Younger adults (M = 308 ms, SEM = 5 ms), and Vertical motion (M = 320 ms, SEM = 5 ms) yields faster responses than Horizontal motion (M = 346 ms, SEM = 8 ms). In addition, we observed a main effect of Deviation (F(9,324) = 3.68, p<0.01), indicating that RT slows when the direction of motion increases its Deviation from the Expected (most probable) direction. This result, depicted in the form of a tuning function in Fig. 3B, parallels the results originally reported by Ball and Sekuler [4]. This suggests that both younger and older adults utilize direction tuning based expectation processes to enhance motion detection abilities. Finally, an Age×Axis interaction was observed (F(1,36) = 3.97, p = 0.05). Similar to the previous ANOVA on probability based expectation, direct comparisons between the Age groups indicate that Older adults respond slower than Younger adults for each Axis of motion (p<0.05, each comparison). However, Older adults expecting Horizontal motion were slower to respond relative to Older adults expecting Vertical motion (t(18) = 3.67, p<0.01), whereas Younger adults showed no such difference (t(18) = 0.49, p = 0.63).

Since Deviation interacted with neither Age nor Axis (each interaction: p>0.2), these results suggest that deviations from the expected direction of motion result in slowed detection performance, regardless of Age or Axis of motion. Hence, the Age×Axis interaction may be interpreted as a change in expectation processes for motion, and not as an age-related decline in processing a specific axis of motion. In support of this assertion, if there were age-based perceptual declines in Horizontal motion processing, detection of Horizontal motion would be slower than detection of Vertical motion regardless of expectation. However, a direct comparison in Older adults between unexpected horizontal (i.e., expected vertical and observed horizontal) and unexpected vertical (i.e., expected horizontal and observed vertical) motion shows slowed RT to unexpected vertical motion (unexpected horizontal: M = 342 ms, SEM = 5 ms; unexpected vertical: M = 380 ms, SEM = 11 ms; t(18) = 3.30, p<0.01). Thus, horizontal motion detection is not always slowed relative to vertical motion detection, ruling out an age-based decline in perceptual processing of horizontal motion. Importantly, the Age×Axis interaction suggests that older adults' expectation for horizontal motion results in slowed RT, regardless of the direction of motion. Together, our findings indicate that horizontal motion processing is not selectively affected by aging, but rather, expectation for horizontal motion produces the older adults' deficient detection performance.

### 2. Ex-Gaussian analysis

**Figure 4:**
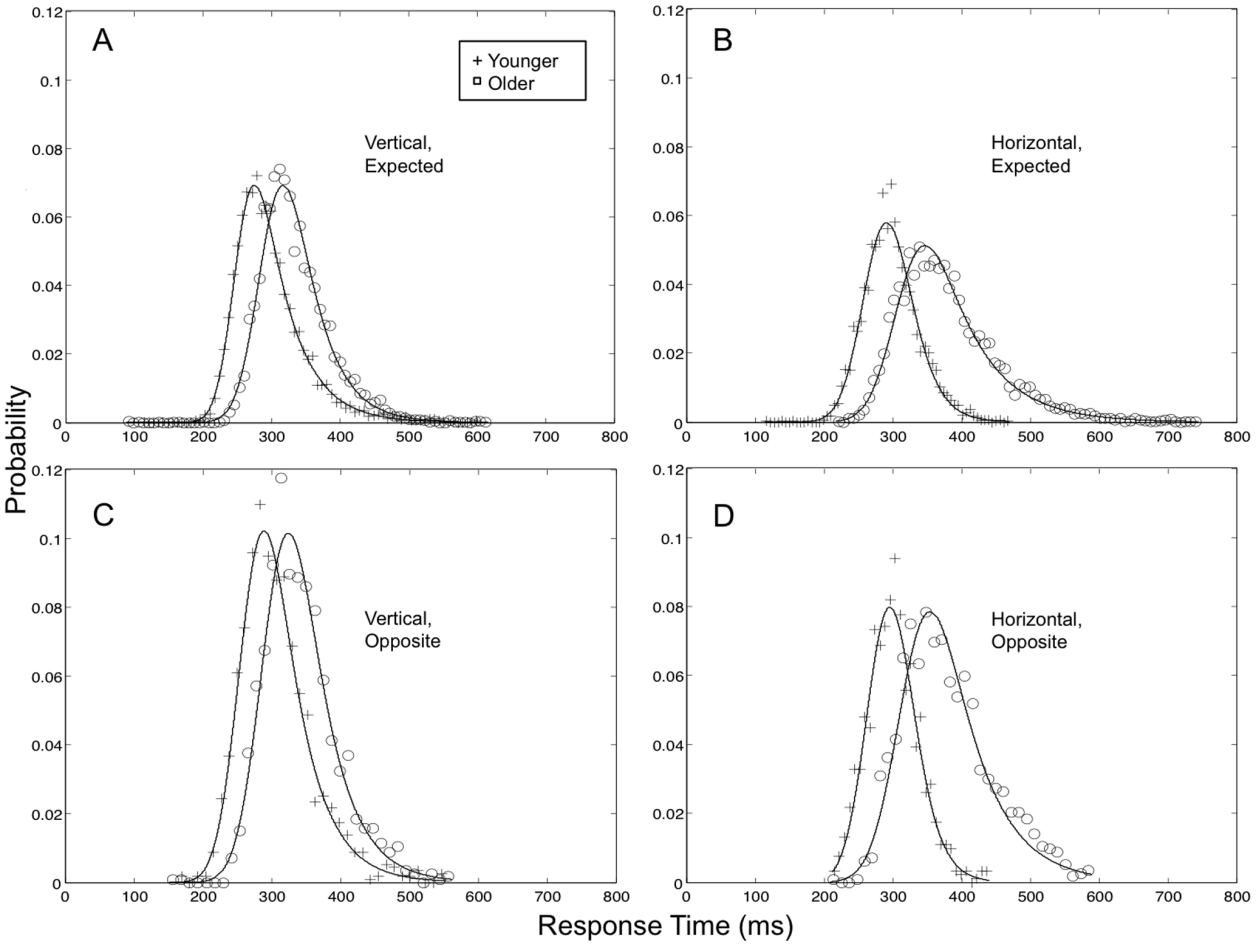
Results of Ex-Gaussian Analysis. Panels A–D show observed RT data from Older (circles) and Younger (pluses) subjects, with the corresponding Ex-Gaussian curves. All data are from the Multidirectional condition. A comparison of performance data following Vertical (Panels A and C) and Horizontal expectations (Panels B and D) shows a rightward shift and spread in the distributions for Older adults (slower and more variable RTs in the Horizontal group) that is not apparent for Younger adults.

To parallel our analysis of mean RTs, an Ex-Gaussian analysis examined the effects of Deviation, Age, and Axis of expected direction (Horizontal or Vertical) for each Ex-Gaussian parameter (μ, σ, and τ). To better assess the effects of direction, this analysis focuses on the Multidirectional condition (refer to Results S1, Figure S1, and Table S1 for the full analysis). A plot of the group-averaged RT distributions and the corresponding Ex-Gaussian functions is presented in Figure 4. Perhaps the most pronounced effect apparent in the figure is a rightward shifting and spreading of the RT distributions for Older adults who were expecting Horizontal motion. This effect is small or absent in Younger adults' distributions, consistent with an Age×Axis interaction in both the location and spread of the RT distributions. Interesting, the effect is absent also in the RT distributions for Older adults when they were expecting Vertical motion. Analysis of the Ex-Gaussian parameter μ showed main effects of Age (Older>Younger; F(1,34) = 26.97, p<0.001), Deviation (Opposite>Expected; F(1, 34) = 13.09, p<0.01), and Axis (Horizontal>Vertical; F(1,34) = 5.90, p<0.05). We also observed an Age×Axis interaction (F(1,34) = 4.32, p<0.05), indicating that the effect of Axis (Horizontal>Vertical) was larger in Older than in Younger adults (t(34) = 2.08, p<0.05). For σ, we observed a main effect of Age (Older>Younger, F(1, 34) = 7.90, p<0.01), and a marginal effect of Deviation (Opposite>Expected, F(1,34) = 4.08, p = 0.05). We again observed an Age×Axis interaction (F(1,34) = 4.44, p<0.05), indicating an effect of Axis (Horizontal>Vertical) that was larger in Older than Younger adults (t(34) = 2.11, p<0.05). For τ, we observed a main effect of Age (Older>Younger, F(1,34) = 14.12, p<0.01). No other τ effects reached significance.

Overall, these analyses confirm that the effects from the previous RT analyses are due to expectation. Furthermore, these results show that within the Multidirectional condition, Age acts to slow the fastest responses in the subjects' RT distributions, producing a shift and increase in spread of the distribution of fast RTs with Age. Finally, the Ex-Gaussian results also show that expectation for horizontal (relative to vertical) motion slows and spreads the distributions of older adults' fastest RTs, though no such effects were observed for younger adults. Together, this extends the previous RT analysis to show that expectation for horizontal motion not only slows detection, but creates more variable responses.

### 3. P1 ERP component

To address the effects of age-related expectation differences on neural activity, ERP analysis focused on the Multidirectional condition, as the Unidirectional condition cannot differentiate expectation from perceptual changes in the Age×Axis interaction for RT. Thus, P1 amplitude and latency data from the Multidirectional condition were submitted to separate ANOVAs, each with Age (Younger, Older), Axis (Horizontal, Vertical), Deviation (Expected, ±22.5°, ±45°, ±67.5°, ±90°, 180°), and Electrode (Left, Right, Central) as factors. However, no main effects or interactions were observed for the P1 amplitude or latency. Thus, neural activity during the P1 does not appear to be involved in the observed age-related RT differences with expectation.

### 4. N1 ERP component

N1 amplitude and latency data from the Multidirectional condition were submitted to separate ANOVAs with Age (Younger, Older), Axis (Horizontal, Vertical), Deviation (Expected, ±22.5°, ±45°, ±67.5°, ±90°, 180°), and Electrode (Left, Right, Central) as factors. The N1 latency exhibited a main effect of Electrode (F(2,72) = 7.13, p<0.01), such that the latency was shorter at Central (M = 170 ms, SEM = 1 ms) electrodes compared to electrodes in the Left (M = 177 ms, SEM = 1 ms) or Right (M = 179 ms, SEM = 1 ms) hemisphere. Furthermore, a 4-way Age×Axis×Deviation×Electrode interaction (F(18,648) = 1.89, p<0.05) was observed. To explore this interaction, separate 3-way ANOVAs were conducted on each Electrode group (Left, Right, Central). Results yielded no main effects or interactions for the Right and Central Electrode groups. However, the Left Electrode group displayed a 2-way Axis×Deviation (F(9,324) = 2.29, p<0.05) and a 3-way Age×Axis×Deviation (F(9,324) = 2.26, p<0.05) interaction. Subsequent analysis of the 3-way interaction utilized separate ANOVAs for each level of the Age factor (Younger, Older). These results elicited no main effects or interactions for the Younger group, yet Older adults yielded an Axis×Deviation (F(9,162) = 3.36, p<0.01) interaction. Post-hoc *t*-tests were conducted between the two Axis of expectation for each direction of motion. Vertical, compared to Horizontal, expectation resulted in a shorter N1 latency when the direction of motion was −45° from the Expected direction, whereas Horizontal, compared to Vertical, expectation resulted in shorter N1 latency when the direction of motion was 180° from the Expected direction (p<0.05, each comparison). Nonetheless, the N1 latency across directions did not follow a similar U-shaped curve as observed in the behavioral data, nor were age-related differences observed for Horizontal motion. Therefore, it is unlikely that the N1 latency reflects age-related changes in expectation-based motion detection.

**Figure 5:**
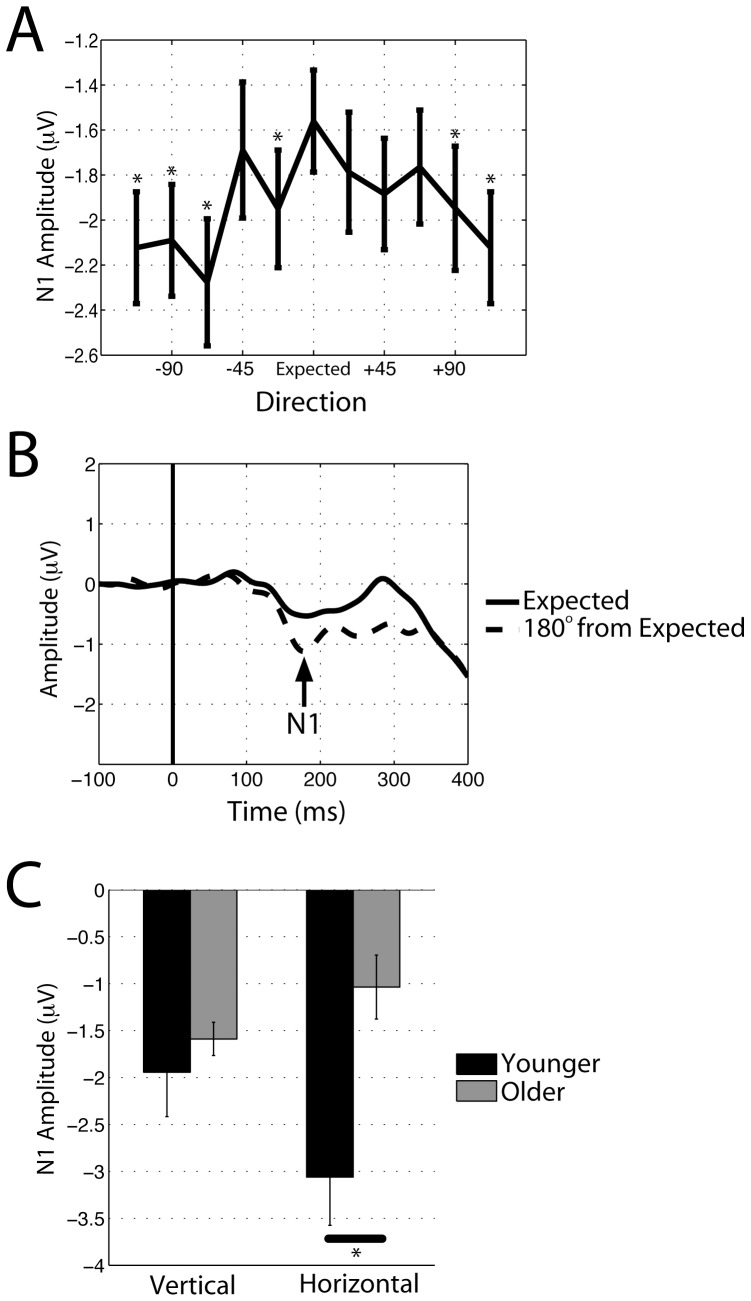
ERP results. (A) Main effect of Deviation indicates increased N1 amplitude with increasing deviation from the Expected direction of motion during the Multidirectional condition. Asterisk indicates p<0.05 (relative to the Expected direction). (B) Example of the ERP waveforms from two directions of motion: Expected and 180° from the Expected direction (averaged over factors Age, Electrode, and Axis). (C) Interaction between Age and Axis shows an age-related change in the N1 amplitude when Horizontal motion is expected. Asterisk indicates p<0.05.

Analysis of the 4-way ANOVA conducted for the N1 amplitude resulted in main effects for Age (F(1,36) = 8.90, p<0.01), Deviation (F(9,324) = 2.34, p<0.05), and Electrode (F(2,72) = 3.69, p<0.05). These results show that Older adults displayed smaller (i.e., less negative) N1 amplitudes (M = −1.31 µV, SEM = 0.07 µV) than Younger adults (M = −2.50 µV, SEM = 0.09 µV), that the Central Electrodes elicited a smaller N1 (M = −1.54 µV, SEM = 0.09 µV) than the Left (M = −2.24 µV, SEM = 0.07 µV) or Right sided Electrodes (M = −1.95 µV, SEM = 0.09 µV), and that the Expected direction produced the smallest N1, which increases in amplitude with increased Deviation from the Expected direction of motion. As illustrated in Figure 5A (see also 5B), the N1 follows a parabolic curve that complements the U-shaped RT tuning curve plotted in Figure 3B. This provides direct support for the neural biasing hypothesis [4]. Furthermore, an Age×Axis (F(1,36) = 4.69, p<0.05) interaction was observed, again similar to behavioral results. Post-hoc *t*-tests showed a reduction in N1 amplitude with age, which only occurs when Horizontal motion was expected (Older vs. Younger, Horizontal: t(18) = 3.29, p<0.01; Older vs. Younger, Vertical: t(18) = 0.70, p>0.49; Fig. 4C). No differences were observed within either Age group when comparing Horizontal to Vertical motion expectation (p>0.1, each comparison). Together, these data provide insight into the neural mechanisms that support expectation-based bias for visual motion in particular directions [3], [4]. Moreover, these results suggest that age-related declines in detection when expecting horizontal motion may stem from changes in early sensory processing, as reflected by the N1 amplitude.

## Discussion

Our study assessed expectation effects in younger and older adults that were based on 1) probability, 2) directional tuning, and 3) axis of motion. Our results indicate that in general, regardless of age, neural and response time measures were consistently modulated by both the probability of motion direction and the magnitude of deviation from the expected direction of motion. We extended the psychophysical findings of Ball and Sekuler [4], and provided substantiation of their neural biasing interpretation: N1 amplitudes followed a U-shaped tuning curve that was similar to RT tuning curves. The established N1 sensitivity to both motion [21] and expectation [23] suggests that expectation-based directional selection occurs during early visual processing stages. Although the forms of these neural and psychophysical curves did not differ with age, older adults were also slower and showed reduced N1 amplitude when they were expecting horizontal motion. This suggests that early visual detection stages are in fact modulated by expectation and selectively diminish in normal aging when subjects expect horizontal motion. Thus, while expectation enhances performance in both younger and older adults, that enhancement is reduced for older adults who were expecting horizontal motion. Together, these results are consistent with recent reports indicating that motion processing mechanisms do not exhibit a general perceptual decline in aging, but rather, are contingent on the type of motion [45], [46], [47]. Here, we extend these previous reports to show that only select types of expectation for motion are altered in aging.

On the surface, one aspect of our findings is paradoxical. When averaged across age and axis of motion, the N1 amplitude decreased for the expected direction of motion, followed by speeded RTs. This is consistent with the idea that reduced N1 amplitudes are associated with faster responding. Yet, when older adults expected horizontal motion, their reduced N1 amplitude was associated with slower responding. One way to interpret this scenario is in the context of the neural signal-to-noise ratio. Over the last 50 years, several hypotheses of cognitive aging have proposed that neural noise throughout the brain accompanies age-related declines in various cognitive tasks [48], [49], [50], [51]. In this neural noise model, a decreased signal-to-noise ratio leads to slowing at information processing stages that generate perceptual representations. Here, increased N1 amplitude may reflect additional neural processing due to a deviation from a (expected) perceptual template or memory trace [52], [53], [54], whereas the N1 is reduced when expectations are fulfilled. However, when older adults expected horizontal motion, their relatively reduced N1 may reflect a decreased signal-to-noise ratio, which might have been insufficient to support proper evaluation of the motion stimuli. In turn, this might have contributed to slowed response times. Although additional research is required to address this intriguing hypothesis, some of our behavioral data suggest that neural noise may underlie the observed age-based changes in expectation. The Ex-Gaussian parameter σ was selectively affected in older adults when horizontal motion was expected. This indicates that response times were more variable in older adults expecting horizontal motion, which would be predicted by the neural noise hypothesis.

Building on the idea that neural noise may underlie these age-based differences, it could be presumed that confidence, and not necessarily expectation, declines in age, which would lead to slower RT. This is especially appealing given that early measures of sensory processes were affected in aging, thereby producing a low-fidelity internal representation of the motion stimuli that would delay detection performance. However, if confidence were the source of the observed aging differences, this would affect both vertical and horizontal motion detection, regardless of the expected direction of motion. Yet, it was observed that older adults expecting horizontal motion exhibited slowed detection performance to all directions of motion that were presented.

Similarly, the neural noise hypothesis does not explain why aging selectively impacts motion detection when expecting horizontal motion. The simplest explanation would be to attribute this selective decline in expectation for horizontal processing to random differences in the group assigned to horizontal motion stimuli as the expected direction. However, the older adults who expected horizontal motion did not differ from the older adults who expected vertical motion in any cognitive (p>0.1, each comparison, uncorrected) or motoric (p>0.6, each comparison, uncorrected) score that was assessed by our battery of 18 neuropsychological tests. Thus, it seems unlikely that differences between the two sub-groups of older adults could account for the age-related expectation deficit for horizontal motion.

An alternative account involves the way in which horizontal motion is processed in the brain. It has been shown that during central fixation, young observers are biased towards vertical rather than horizontal motion when viewing a bistable stimulus that supported both directions [13], [14], [15]. This effect has been attributed to horizontal motion requiring interhemispheric integration that may introduce delays in transcallosal processing or signal degradation, whereas vertical motion relies on intrahemispheric processing whose efficiency makes it the preferred percept. The difference in inter- and intrahemispheric motion processing most likely reflects the fact that vertical motion does not cross the central meridian, whereas horizontal motion utilizes both hemifields to create a stable percept of motion. The additional distance neural signals must travel during interhemispheric communication could result in age-related declines selective for horizontal motion processing, as the structural integrity of white matter tracts is known to degrade in aging and is predictive of age-related declines in processing speed [16], [17]. Data from commissurotomized patients indicate that subcortical structures mediate interhemispheric communication between bilateral V5/hMT [55], [56], and recent research in normal adults suggests the pulvinar nucleus of the thalamus is an anatomical hub for integrating horizontal motion processes [57]. Although it is unclear what neural regions may underlie the directional tuning afforded by expectation processes, it is known that the prefrontal cortex is involved in forming expectations [58], [59], [60] that bias motion sensitive cortex (V5/hMT+)[5], [22] and this area has direct connections to the pulvinar nucleus [61], [62], [63]. Interestingly, older adults display gray matter atrophy in both prefrontal and thalamic regions, including the pulvinar nucleus [64], [65], [66], which may underlie the expectation-based deficit in horizontal processing. Yet, this remains to be directly addressed. If true, it could be consistent with the neural noise hypothesis if it were also shown that vertical motion processing is biased by expectations via a corticocortical connection. Thus, according to the neural noise hypothesis, degradation of expectation signals and/or an increase in noise would be most prominent in the longer, more complex, corticothalamocortical pathway that is needed for biasing horizontal motion processing,

Another intriguing possibility, although not necessarily distinct from the supposition that expectations for horizontal and vertical motion rely on differential neural pathways, is that gravity may play a role in retaining the ability to expect vertical motion throughout the lifespan. Internal representations of gravity are biased towards the observer's body orientation, such that when an observer lies on their side, the expected effects of gravity are biased towards their subjective vertical axis of motion, although gravity acts on the subjective horizontal axis [67], [68]. These effects are thought to rely on multisensory estimates of gravity [69]. Thus, forming expectations for the effects of gravity on a daily basis may serve to stave off age-related changes for expectations of vertical (but not horizontal) motion.

It should be noted that the *Axis* factor (vertical or horizontal) was implemented between, not within, subjects due to time constrains. In order for each participant to be assessed on both vertical and horizontal expectation, an additional 90 minutes of testing would have been required, which most likely would have introduced fatigue effects. Although the current design does not permit an assessment of relative changes in expectation within each participant, we believe a repeated measures design would only confirm, and possibly strengthen, our findings of an age-related decline in motion detection when expecting horizontal motion.

The current study's results may help explain why older adults are more prone to traffic accidents at intersections, turning, and changing lanes [70], [71]. Each of these scenarios invokes expectations for horizontal motion, which we have shown decrease motion detection ability in older adults in all directions. Although speculative, it is intriguing that the cause of accidents in older adults is typically attributed to estimation errors in the motion of another vehicle while they are moving horizontally (e.g., turning at an intersection), whereas younger adults are more prone to accidents involving only their car and are less likely to be caused by motion estimation error [71]. Moreover, it is interesting to note that older adults are less prone than younger adults to be involved in an accident due to driver fatigue, inclement weather, and high speeds [70], which could indicate that age-based differences in driving performance may not be solely attributed to more general declines in attention or sensory-motor synchronization. Nonetheless, additional research will be required to determine whether age-related expectation-based declines in motion processing actually undermine driving performance such as the detection of cross-traffic or pedestrians.

## Conclusions

Our results suggest expectation can bias responses in motion selective neural regions, leading to more efficient motion detection. However, during early stages of visual processing, older adults exhibit a decline in ability to utilize expectation for horizontal motion in order to influence neural activity. This results in deficient detection performance. Together, our results suggest that older adults do not display a generalized decline in modulating directional selectivity, but rather, show differential expectation abilities that vary with axis of motion.

## Supporting Information

Figure S1 **Observed RT data from Older (circles) and Younger (pluses) subjects, with the corresponding Ex-Gaussian curves.** All data are from the Expected direction of motion. A comparison of the Vertical conditions (Panels A and C) with the Horizontal conditions (Panels B and D) shows a rightward shift and spread in the distributions for Older adults (slower and more variable RTs in the Horizontal group) that is not apparent for Younger adults.
(TIFF)

Results S1 **Ex-Gaussian analysis of performance data.**
(DOC)

Table S1 **Mean parameter values of the Ex-Gaussian distribution for Younger and Older subjects expecting Horizontal or Vertical motion.**
(DOCX)
