# Supplementary figures and images for: Age-Related Changes in Expectation-Based Modulation of Motion Detectability
Source: PLoS One. 2013 Aug 9;8(8):e69766. doi: 10.1371/journal.pone.0069766 (PMC3739821; doi:10.1371/journal.pone.0069766)

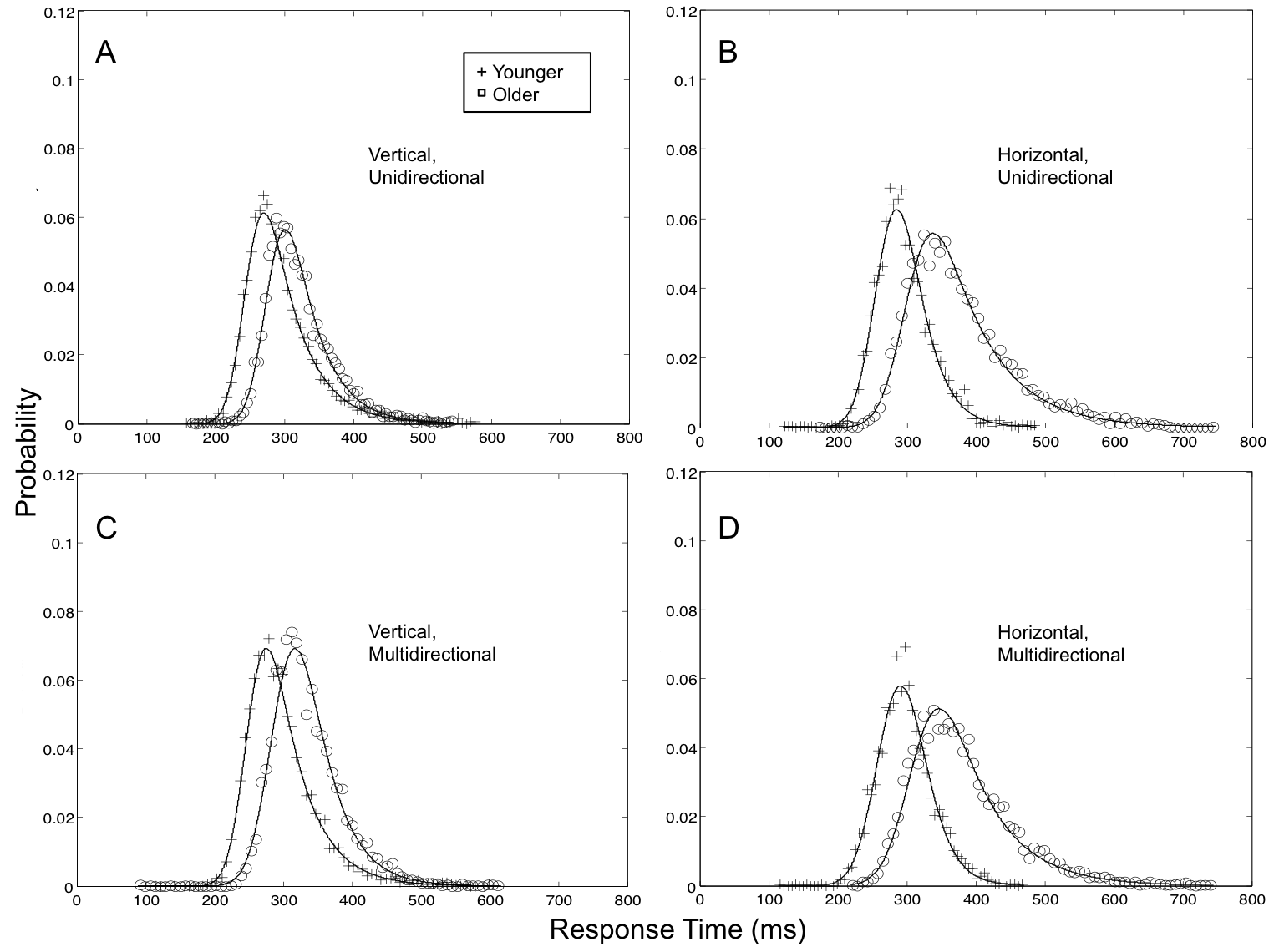

Supplement: Figure S1 — Observed RT data from Older (circles) and Younger (pluses) subjects, with the corresponding Ex-Gaussian curves. All data are from the Expected direction of motion. A comparison of the Vertical conditions (Panels A and C) with the Horizontal conditions (Panels B and D) shows a rightward shift and spread in the distributions for Older adults (slower and more variable RTs in the Horizontal group) that is not apparent for Younger adults. (TIFF) [file pone.0069766.s001.tiff]
