# Supplementary material for: Age-Related Changes in Expectation-Based Modulation of Motion Detectability
Source: PLoS One. 2013 Aug 9;8(8):e69766. doi: 10.1371/journal.pone.0069766 (PMC3739821; doi:10.1371/journal.pone.0069766)
Supplement: Results S1 — Ex-Gaussian analysis of performance data. (DOC) [file pone.0069766.s002.doc]

**Supporting Results**

Ex-Gaussian analysis

*Analysis of the Expected Direction*

As in our analysis of mean RTs, we analyzed the effects of Age, Probability, and Axis of the expected direction, using only data obtained on trials on which the Expected direction was presented. Mean parameter values are presented in Table S1, and a plot of the group-averaged RT distributions and corresponding Ex-Gaussian functions for responses to the expected direction is presented in Figure S1.

A number of effects are apparent in Figure S1. First, each panel shows an age-related shift in the RT distributions: the distributions for Older adults are consistently located rightward of the Younger adults’ distributions. Second, RT distributions appear to be more variable overall for Older adults. This is especially apparent in the right tails of the distributions: Older adults show greater right skew, consistent with a shift in the Ex-Gaussian model’s exponential component. This indicates slowing in the longest RTs with Age, which parallels the slowing of the fastest responses with Age suggested by the rightward shift. Finally, it appears that the skew effect is magnified when Older adults expect Horizontal motion. This is particularly noteworthy, as Younger adults do not show any such effects of Axis of expected motion (Fig. S1)[[1]](#footnote-2). Thus, it appears that the Age x Axis effect uncovered in our analysis of mean RTs is driven in part by an Age-dependent effect of expectation on responses occurring later in time following stimulus onset.

*Analysis of μ*

To verify the effects apparent in Figure S1, we conducted a 2 (Age, between) x 2 (Axis, between) x 2 (Probability, within) mixed ANOVA, conducted separately for each of the three Ex-Gaussian parameters. The analysis of *μ* showed significant main effects of Age (Older > Younger), *F(1, 34) = 33.54, p<.001,* Axis (Horizontal > Vertical), *F(1, 34) = 6.55, p<.05*, and Probability (Multidirectional > Unidirectional), *F(1, 34) = 34.76, p<.001.* These results demonstrate that the factors of Age, Axis, and Probability shift the Gaussian component along the x-Axis, as illustrated in Figure S1. We also found a significant interaction between Age and Probability, *F(1, 34) = 6.11, p<.001*, and a marginal interaction between Age and Axis, *F(1, 34) = 3.85, p = .058.* Follow-up tests revealed that the effect of Probability (Multidirectional > Unidirectional) was more pronounced in Older than in Younger subjects, *t(36) = 2.53, p<.05*. The Age x Axis effect arose from the fact that Younger subjects did not show a significant effect of Axis, while Older subjects showed larger values of *μ* when expecting the Horizontal Axis of motion, *t(18) = 3.45, p<.01.* This can be seen in Figure S1 as a rightward shift of the distributions’ peak for older adults, from the left to right panels.

*Analysis of σ*

For the analysis of *σ*, we again found main effects of Age (Older > Younger) and Probability (Multidirectional > Unidirectional), *F(1, 34) = 9.01, p<.01* and *F(1, 34) = 10.17, p<.01*, respectively. We also found a significant interaction between Age and Axis, *F(1, 34) = 5.73, p<.05*. As we saw previously for *μ,* only Older adults showed an effect of Axis (Horizontal > Vertical), *t(18) = 2.55, p<.05*. These analyses show that Age and Probability produce changes in the variance of the Gaussian component of the RT distributions, and that Older adults expecting Horizontal motion were more variable in their fastest responses than were Older adults expecting Vertical motion.

*Analysis of τ*

The analysis of the *τ* parameter produced a main effect of Age (Older > Younger), *F(1, 34) = 11.38, p<.01,* as well as significant interactions between Probability and Axis, and between Age and Axis, *F(1, 34) = 5.25, p<.05*, and *F(1, 34) = 5.59, p<.05*, respectively. Follow-up tests showed that while expecting Vertical motion did not show an effect of Probability, Horizontal motion expectation produced slightly smaller values of *τ* in the Unidirectional condition, *t(17) = 2.15, p<.05.* As we saw for the *σ* parameter*,* the Age x Axis interaction was due to the fact that only Older adults showed an effect of Axis (Horizontal > Vertical), *t(18) = 2.55, p<.05.* This indicates that the exponential component of the RT distributions was shifted rightward in Older subjects expecting Horizontal rather than Vertical motion. This verifies the effect we noted in visual inspection of Figure S1: Older adults show an effect of expectation on responses that occur relatively late following stimulus onset.

1. Note the peaks of the Horizontal distributions for Younger adults are lower than in panels A and C, due to the removal of the two outlying subjects in the former condition. This affects the display for the group data, but not our analyses, which were conducted on the individuals’ best-fitting Ex-Gaussian parameters. [↑](#footnote-ref-2)
