# Supplementary material for: Age-Related Changes in Expectation-Based Modulation of Motion Detectability
Source: PLoS One. 2013 Aug 9;8(8):e69766. doi: 10.1371/journal.pone.0069766 (PMC3739821; doi:10.1371/journal.pone.0069766)
Supplement: Table S1 — Mean parameter values of the Ex-Gaussian distribution for Younger and Older subjects expecting Horizontal or Vertical motion. (DOCX) [file pone.0069766.s003.docx]

**Table S1**

|  |  | Horizontal | | Vertical | |
| --- | --- | --- | --- | --- | --- |
|  | Parameter | Older | Younger | Older | Younger |
| Multidirectional Condition,  Expected Direction | *μ* | 327.11 | 269.53 | 294.22 | 265.31 |
|  | *σ* | 33.69 | 21.44 | 24.57 | 23.03 |
|  | *τ* | 52.60 | 28.52 | 39.42 | 33.21 |
| Multidimentional Condition,  Opposite Direction | *μ* | 337.52 | 279.55 | 298.81 | 278.20 |
|  | *σ* | 36.25 | 24.33 | 28.38 | 26.45 |
|  | *τ* | 42.69 | 21.77 | 43.24 | 29.37 |
| Unidirectional Condition | *μ* | 314.58 | 264.97 | 284.97 | 260.96 |
|  | *σ* | 28.13 | 19.76 | 22.34 | 21.55 |
|  | *τ* | 60.92 | 30.64 | 36.91 | 33.55 |
